# Identification of volatiles released by diapausing brown marmorated stink bug, *Halyomorpha halys* (Hemiptera: Pentatomidae)

**DOI:** 10.1371/journal.pone.0191223

**Published:** 2018-01-17

**Authors:** Laura J. Nixon, William R. Morrison, Kevin B. Rice, Eckehard G. Brockerhoff, Tracy C. Leskey, Filadelfo Guzman, Ashot Khrimian, Stephen Goldson, Michael Rostás

**Affiliations:** 1 Bio-Protection Research Centre, Lincoln University, Lincoln, Canterbury, New Zealand; 2 Better Border Biosecurity Collaboration, Christchurch, New Zealand; 3 USDA-ARS Center for Grain and Animal Health, Manhattan, KS, United States of America; 4 USDA Appalachian Fruit Research Station, Kearneysville, WV, United States of America; 5 Scion (New Zealand Forest Research Institute), Christchurch, New Zealand; 6 USDA-ARS, NEA, IIBBL, 10300 Baltimore Blvd, Beltsville, MD, United States of America; 7 AgResearch Ltd, Christchurch, New Zealand; Korea Research Institute of Bioscience and Biotechnology, REPUBLIC OF KOREA

## Abstract

The brown marmorated stink bug, *Halyomorpha halys*, is an agricultural and urban pest that has become widely established as an invasive species of major concern in the USA and across Europe. This species forms large aggregations when entering diapause, and it is often these aggregations that are found by officials conducting inspections of internationally shipped freight. Identifying the presence of diapausing aggregations of *H*. *halys* using their emissions of volatile organic compounds (VOCs) may be a potential means for detecting and intercepting them during international freight inspections. Headspace samples were collected from aggregations of diapausing *H*. *halys* using volatile collection traps (VCTs) and solid phase microextraction. The only compound detected in all samples was tridecane, with small amounts of *(E)-*2-decenal found in most samples. We also monitored the release of defensive odors, following mechanical agitation of diapausing and diapause-disrupted adult *H*. *halys*. Diapausing groups were significantly more likely to release defensive odors than diapause-disrupted groups. The predominant compounds consistently found from both groups were tridecane, *(E)*-2-decenal, and 4-oxo*-(E)-*2-hexenal, with a small abundance of dodecane. Our findings show that diapausing *H*. *halys* do release defensive compounds, and suggest that volatile sampling may be feasible to detect *H*. *halys* in freight.

## Introduction

*Halyomorpha halys*, commonly known as the brown marmorated stink bug, has emerged as a severe agricultural and urban pest in the USA [1]. Originally from China, Korea, Japan, and Taiwan, this invasive species has spread across 43 states of the US and two provinces in Canada since it was first officially detected in Pennsylvania in 2001 [1]. As of September 2017, *Halyomorpha halys* have successfully invaded and established in 43 states of the USA, Canada, and nine European countries (Italy, France, Hungary, Switzerland, Germany, Liechtenstein, Greece, Serbia, and Romania), with recent incursions reported in Russia, Georgia, and Bulgaria [1–7]. Europe’s temperate climate is suitable for its success [8]; the models developed by Zhu et al. [8] identify regions within the latitudes 30°–50° as high risk from *H*. *halys* invasion. The species has also recently been reported in Chile, it’s first Southern Hemisphere invasion [9].

Like many heteropterans, *H*. *halys* spends the winter months in diapause [10]. In the United States, the bugs begin to disperse in late summer to overwintering sites. Large numbers aggregate at suitable sites and go into diapause from early to mid-October with spring emergence commencing in April. Typical overwintering sites include dry, protected structures including human-made dwellings, beneath the bark of dead and standing trees [11], as well as dry, elevated locations [10]. This behavior causes problems for border biosecurity in other countries, as *H*. *halys* is an adept hitchhiker and overwintering aggregations can be exported in personal effects or vehicle shipments [12]. Conditions caused by shipment, such as temperature increases, photoperiod shifts, and constant movements, have the potential to disrupt the bugs’ diapausing state. This is particularly problematic, because pheromone-baited traps that were developed for monitoring *H*. *halys* during the growing season are ineffective against overwintering populations [13]. The significance of diapausing *H*. *halys* as a biosecurity risk to the international community makes it prudent to investigate alternative measures to detect *H*. *halys*, including the chemical emissions and behaviors of this species in diapause and when diapause becomes disrupted. If a method for detection of emissions of volatiles from *H*. *halys* could be developed, then aggregations of *H*. *halys* in international freight shipments could be detected and treated, thereby preventing arrival and establishment post-border. Volatile organic compounds (VOCs) can be continuously released by organisms. Moreover, many heteropterans also release defensive VOCs usually consisting of tridecane and at least one *(E)*-2-aldehyde, whether it be the 6-, 8-, or 10-carbon aldehyde. Aldehydes are the odor compounds for which “stink” bugs are named [14]. The question of whether diapausing populations of *H*. *halys* release detectable VOCs, or show such a defensive reaction, has not been previously studied. Thus, it is necessary to first establish if VOCs can be detected from aggregations of diapausing populations, and whether this defensive reaction is exhibited by diapausing bugs. This contribution discusses an emissions profile found from diapausing and diapause-disrupted adults of *H*. *halys*, both at rest and when agitated.

## Methods and materials

### Field samples of *H*. *halys*

For the purposes of this study, cohorts of naturally diapausing wild adult *H*. *halys* were used. Simulated overwintering sites, i.e., wooden shelters, as described by Bergh et al. [15], were deployed in September 2015, just prior to dispersal to potential overwintering sites. Shelters were deployed at an organic farm (Redbud Farm) in Inwood, WV (39°23'41.49"N, 78° 4'39.84"W), at Mount Weather, VA (39° 3'43.77"N, 77°53'29.65"W), Boonsboro, MD (39°30'20.55"N, 77°44'34.95"W), and Gerrardstown, WV (39°24'22.19"N, 78° 5'54.68"W).

Thereafter, the shelters were collected in early November 2015 after diapause onset and maintained in a dark unheated shed at USDA-ARS Kearneysville, WV (39°21'18.69"N, 77°52'40.71"W), from November 2015 to March 2016 under ambient temperature conditions (mean ± SE: 6.3 ± 0.03°C). Adults were then utilized based on two relevant biological regimes. These included inactive, diapausing individuals that remained aggregated and diapausing individuals that had become active, i.e., foraging, feeding, and/or reproducing, due to favorable abiotic conditions. To evaluate these two potential conditions, adults described as “diapausing” were taken directly from shelters retrieved from the unheated shed and immediately exposed to the relevant experimental conditions. Adults referred to as “diapause-disrupted” were also taken from shelters in the unheated shed, but subsequently exposed to long photoperiod (16:8 L: D), higher temperatures (24.4 ± 0.2°C), and supplied with food (carrots, sundried tomatoes, and sunflower seeds) *ad libitum* for at least 2 weeks, to begin to break diapause and induce foraging behavior.

### VOC emission by diapausing *H*. *halys*

**Fig 1:**
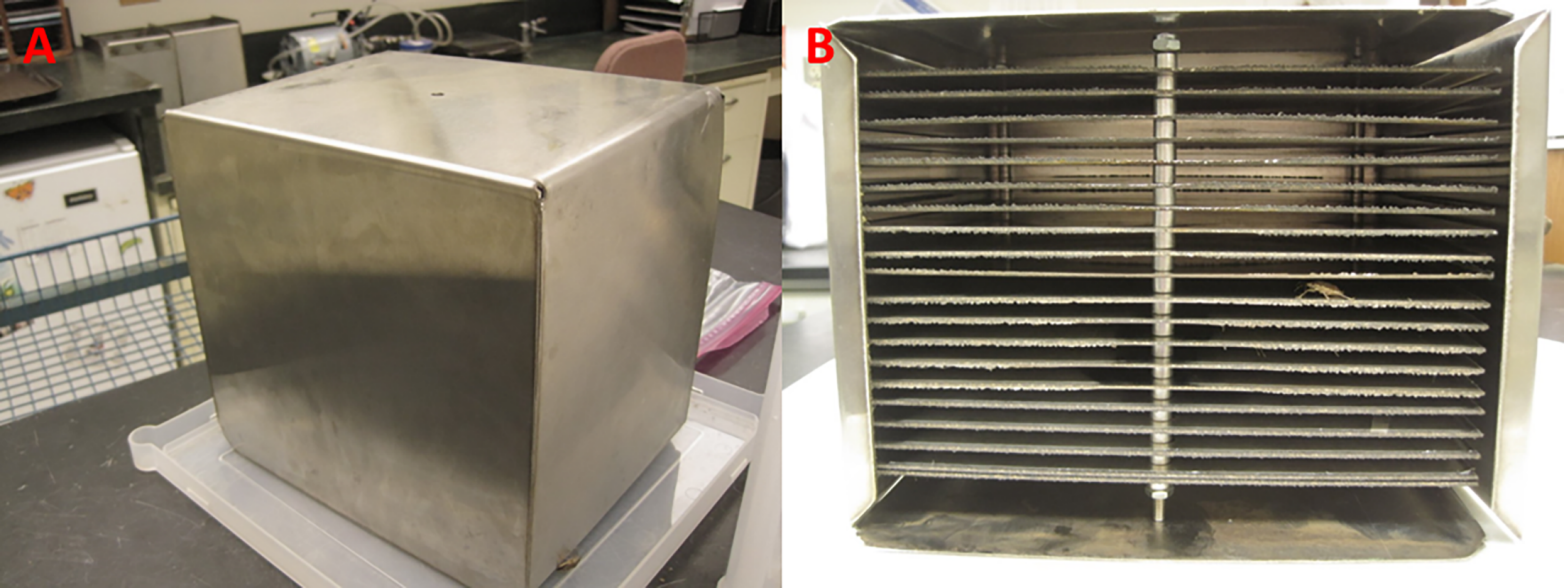
**A**) Photograph of metal sampling box exterior. **B**) Photograph of metal sampling box interior. Credit: Torri Hancock (USDA-ARS, AFRS).

To establish whether there is a sufficient VOC emission profile released by non-agitated bugs, naturally diapausing aggregations of *H*. *halys* were resettled from the wooden shelters into metal shelters (n = 7) and left outside in a darkened shed under ambient temperature and relative humidity. The metal shelters (18 × 22 × 20.5 cm, H × L × W) mimicked the design of the already established wooden shelters as overwintering locations for *H*. *halys* [15]. A total of 10, ~2 mm wide metal sheets (22 × 19.5 cm, L × W) were spaced 9 mm apart in half of the space available within the box and affixed in place with three screws running the length of the box and a series of nuts. Two metal lips on the shelter box overhung the metal sheets by 1.1 cm to prevent the internal assembly from loosening (See Fig 1A and 1B).

The resettling process was conducted by taking 68 adult *H*. *halys* (2.4M: 1F, this ratio was taken from preliminary observations of wild *H*. *halys* settling into overwintering shelters) from the diapausing populations in the wooden shelters and placing them in cages containing the metal shelter. This cage was placed in a temperature controlled room (24.4 ± 0.2°C) with light exposure for 12 hr; this allowed the bugs to become mobile enough to crawl up into the metal shelter, but was not extended enough to disrupt diapause. The critical cue to terminate *H*. *halys* diapause has been shown to be 13.5 hr daylight, this needs to be over an extended period of time to trigger any real disruptions [16, 17]. The metal shelters containing populations of *H*. *halys* were then maintained in a dark outdoor shed under ambient temperature conditions (6.3 ± 0.03°C) for seven days before VOC sampling was performed. This procedure caused minimal disruption to the bugs.

Resettling of the adults into the metal shelters was necessary to eliminate additional background chemical noise, which was an inherent problem for the wooden shelters in which overwintering *H*. *halys* are normally maintained. The headspace of the metal shelters, containing *H*. *halys* that had not undergone any mechanical agitation, were sampled using two methods. First, headspace compounds were collected with a portable battery-operated air pump (PAS-500, Spectrex, CA, USA). Air from the shelters was pulled through a volatile collection trap (VCT) containing 30 mg of Super-Q (Analytical Research Systems, FL, USA) at a rate of 400 ml/min for 2 hr. The VOCs were extracted from VCTs using 250 μl of dichloromethane (DCM). Secondly, solid phase micro-extraction (SPME) headspace samples were collected using 100 μm polydimethylsiloxane (PDMS) fibers with a sampling time of 18 hr. PDMS fibers were chosen as they are recommended for sampling volatile compounds with molecular weight 60–275; the previously mentioned defensive compounds fall within this weight range. Fibers were conditioned at 230°C in a GC injection port for 15 min prior to sampling. Blank control samples were taken from an empty metal shelter using both sampling techniques, and analyzed alongside the relevant samples to eliminate background volatiles.

### Olfactory detection of mechanically agitated *H*. *halys*

We evaluated whether the disposition to emit VOCs differed depending on the bugs’ biological state. To cause a significant level of disturbance, diapausing and diapause-disrupted *H*. *halys* were held in groups of three in 36 ml glass tubes and shaken vigorously by hand for 1 min. This procedure was chosen as a first step to obtain the highest probability of releasing VOCs and as a prerequisite for further experiments that will establish VOC emission under conditions experienced during freight transportation. After 1 min, the experimenter determined if odors were detectable by human olfaction; the same experimenter was involved in this procedure to eliminate observer variation. A total of 25 replicates were completed for each bug condition, and a chi-squared test for independence was performed to assess *H*. *halys* defensive response in relation to diapause state.

### VOCs from mechanically agitated *H*. *halys*

Some mechanically agitated groups of *H*. *halys* were found to release defensive compounds during the olfactory detection experiment. Where this occurred, representative headspace samples were taken from both diapausing (n = 8) and diapause-disrupted (n = 6) *H*. *halys*. Headspace compounds were collected using the VCT method described above, with air from the 36 ml glass tube containing three bugs being sampled for 10 min. The VOCs were extracted from VCTs using 250 μl of DCM with 200 ng/μl tetralin (Sigma-Aldrich, Australia) as an internal standard. A control blank was taken using the same apparatus and extraction technique.

#### Chemical standards

Quantitative calibration standards of 2, 10, 20, 100, and 200 ng/μl were made from *(E)*-2-octenal, *(E)*-2-decenal, and tridecane (all >94%, Sigma-Aldrich, Australia) diluted using dichloromethane. All calibration standards also contained tetralin (IS) added from a 10 μg/μl stock. *(E)*-2-octenal, *(E)*-2-decenal, and tridecane quantities from *H*. *halys* headspace samples were calculated using calibration linear equations. 4-Oxo-(*E*)-2-hexenal was prepared from 2-ethylfuran following Moreira and Millar [18]. To determine whether differences in defensive compound quantity between diapausing and diapause-disrupted H. halys were significant, non-parametric Mann-Whitney U two-tailed tests were performed, following Shapiro-Wilk tests for normality.

### Gas chromatography–mass spectrometry

GC-MS analysis was performed on an Agilent Technologies 7890A gas chromatograph coupled with 5975c mass selective detector with an HP-5MS column (30 m x 0.25 mm I.D. x 0.25 μm film), and He as an inert gas (located at USDA-ARS, Beltsville, MD). The spectra were obtained in electron-impact (EI) ionization mode at 70 eV. Splitless injections of 1 μl at an injection temperature of 250°C using Agilent Technologies 7683B autoinjector and 7683 autosampler. The SPME injection sampling time was 3 min. The GC was operated at a column flow of 0.9 ml/min. The temperature program started at 40°C for 7 min, followed by ramping 6°C/min until a final temperature of 230°C was reached and held for 5 min. The mass spectrometer was simultaneously run in total ion count mode, with a scanning range 25–550 *m/z*, and selected ion mode, detecting ions at 29, 41 and 55 *m/z* for *(E)-*2-octenal, 43, 55, and 70 *m/z* for *(E)-*2-decenal, and at 43, 57, and 71 *m/z* for tridecane. For samples and standards containing tetralin, ions at 91, 104, and 132 *m/z* were also detected.

## Results and discussion

### Compounds detected from aggregations of diapausing *H*. *halys*

Headspace samples collected from undisturbed metal shelters (n = 7) using trapping filters, were found to contain predominantly tridecane (89.7 ± 6.7% abundance of total compounds detected) in all samples; *(E)*-2-decenal (0.9 ± 0.9%) was detected in one sample, and decanal (9.4 ± 6.8%) in three samples. When SPME fibers were used, all samples contained tridecane (88.6 ± 3.0%), and six contained *(E)*-2-decenal (7.4 ± 2.3%). This suggests that aggregations of diapausing *H*. *halys* produce, and possibly passively leak, these compounds over time even in the absence of any disturbance [19]. This is supported by the findings of Baldwin et al. [20], who reported that 70% of the total volatiles emitted by active season, non-agitated adult *H*. *halys* consisted of tridecane and *(E)*-2-decenal. The minor compounds that were detected in our samples, all at <5% abundance, were dodecane (2 samples), decanal (4 samples), *(E)*-2-decenyl acetate (1 sample), a 13C unknown (2 samples), and pentadecane (1 sample). This profile is not specific to *H*. *halys*, as tridecane is a commonly found VOC, reported in headspaces of treated wood, floral scent mixture, and numerous insect species [21–23]. All other VOCs were found inconsistently in low abundance.

### Effect of physiological state on VOC emission

The results from the agitation tests showed that diapausing and diapause-disrupted groups both released defensive odor but not 100% of the time in response to the same mechanical agitation. Further, there were differences between the two groups of bugs: 72% of the diapausing groups released the odor, versus 40% of the diapause-disrupted groups. Thus, diapause significantly affected *H*. *halys’* defensive odor response (X^2^ = 5.195, df = 1, *P* = 0.023). A possible explanation for the higher responsiveness amongst the diapausing bugs could be related to a lack of alternative defensive options; whereas, diapause-disrupted bugs have greater mobility potential [24] enabling them to escape before resorting to chemical defenses. Pentatomids have been shown to prioritize mobility as an escape strategy over the use of defensive chemical response to tactile agitation [25]. From a practical point of view, the observation that diapausing *H*. *halys* have been shown to release defensive VOCs indicates a potential for chemical detection of large aggregations for the purpose of detection and interception purposes in trade pathways.

The VOC blends from diapausing and diapause-disrupted bugs were very similar (Table 1). The predominant component, in terms of abundance, emitted by both groups in response to agitation was tridecane, with three minor components consisting of other *n*-alkanes (C_10_ –C_14_) (Table 1). This homologous series has been commonly reported as a primary component of defensive secretions in pentatomid species, with tridecane predominating. In general, *n*-alkanes are common within heteropteran scent glands [21, 26, 27].

**Table 1:** Compounds released by agitated diapausing (n = 8) and agitated diapause-disrupted (n = 6) adult *Halyomorpha halys*. ‘Compound present’ indicates the proportion of bug groups that released the compound. ‘Percentage of total’ shows the proportion of the compound in relation to the total blend. Amount emitted per bug is given as mean ± SE.

	Diapause			Diapause-disrupted		
Compound	Compound present [%]	Percentage of total [%]	Emission [μg] per bug	Compound present [%}	Percentage of total [%]	Emission [μg] per bug
Tridecane	100	53.1	41.7 ± 11.8	100	56.5	43.4 ± 13.6
*(E)-*2-decenal	100	21.4	18.2 ± 4.2	100	20.3	19.2 ± 5.2
4-oxo*-(E)-*2-hexenal	100	20.6	15.8 ± 6.3*	100	20.3	18.1 ± 5.7*
Dodecane	100	2.6	1.5 ± 0.6*	100	2.0	2.0 ± 0.7*
*(E)-*2-octenal	37.5	0.2	0.8 ± 0.07	50	0.3	0.7 ± 0.17
*(E)-*2-decenyl acetate	37.5	0.2	0.06 ± 0.06*	50	0.6	1.0 ± 0.8*
Undecane	12.5	2.0	0.1 ± 0.06*	16.7	<0.1	0.06 ± 0.06*
Tetradecane	n.d.	-	-	16.7	<0.1	0.04 ± 0.04*

*Estimated using ratio of compound to internal standard, assuming a response ratio of 0.32:1;

n.d. = not detected

Aldehydes produced by stink bugs have been well documented ever since Tsuyuki et al. [28] confirmed them as major components produced by both pentatomids and coreids. Of these compounds *(E)*-2-hexenal, *(E)-*2-octenal, and *(E)*-2-decenal are the most commonly reported within pentatomids. Typically, *(E)*-2-decenal appears as a major component alongside tridecane [29]. Of these, *(E)*-2-decenal was the only compound consistently released by agitated *H*. *halys*. *(E)*-2-octenal was detected in less than half of the samples as a minor component, and *(E)*-2-hexenal found not at all, similarly described by Aldrich [21].

**Fig 2:**
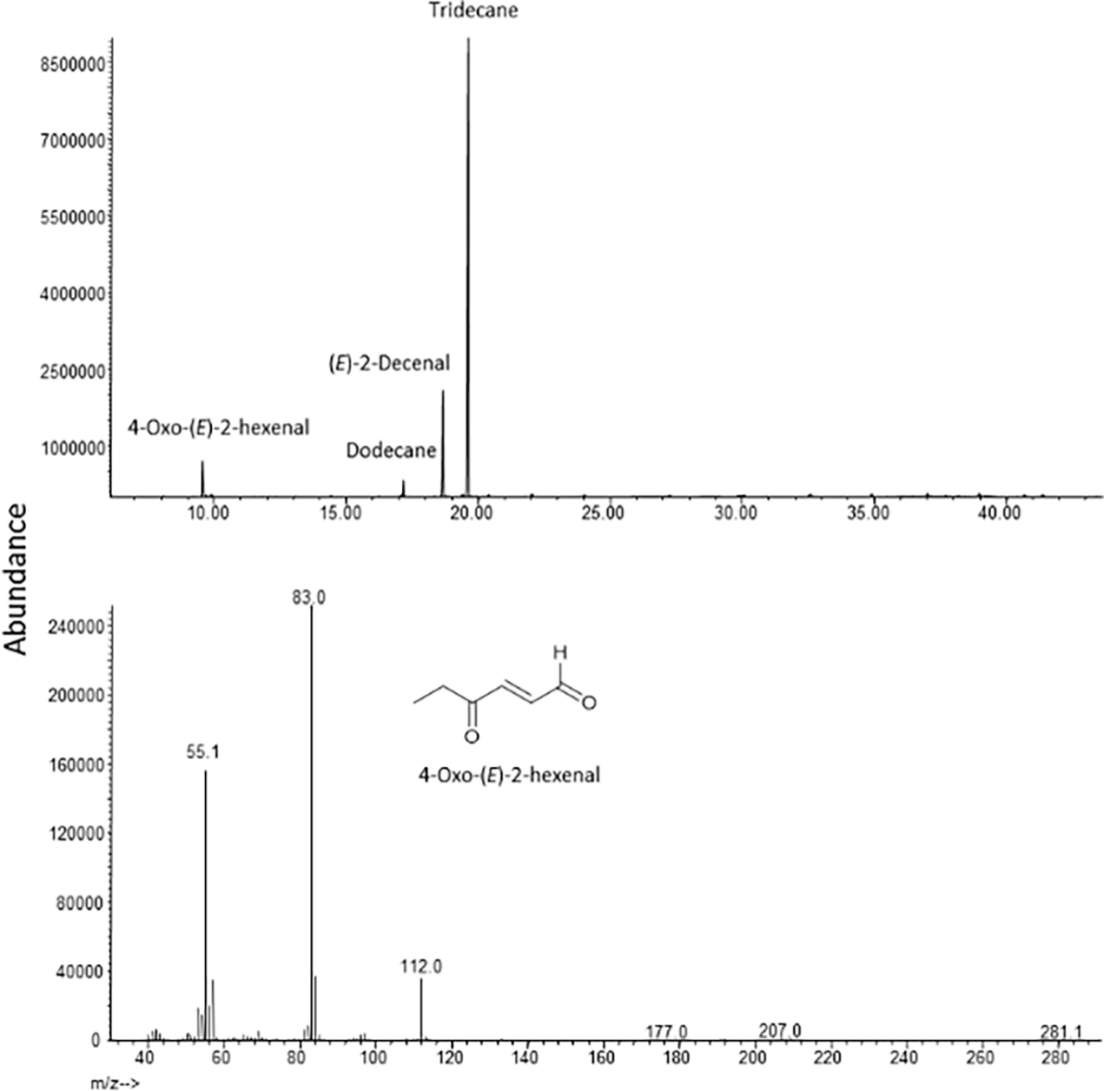
**A)** GC-MS total ion chromatogram of aeration extract in DCM collected from a group of 10 agitated, diapausing *H*. *halys* on a HP-5MS. **B)** Mass spectrum of the peak at 9.58 min identified as 4-oxo-(*E*)-2-hexenal. Compounds were identified by comparing GC retention times and mass spectra with those of standards (see also Fig 3).

**Fig 3:**
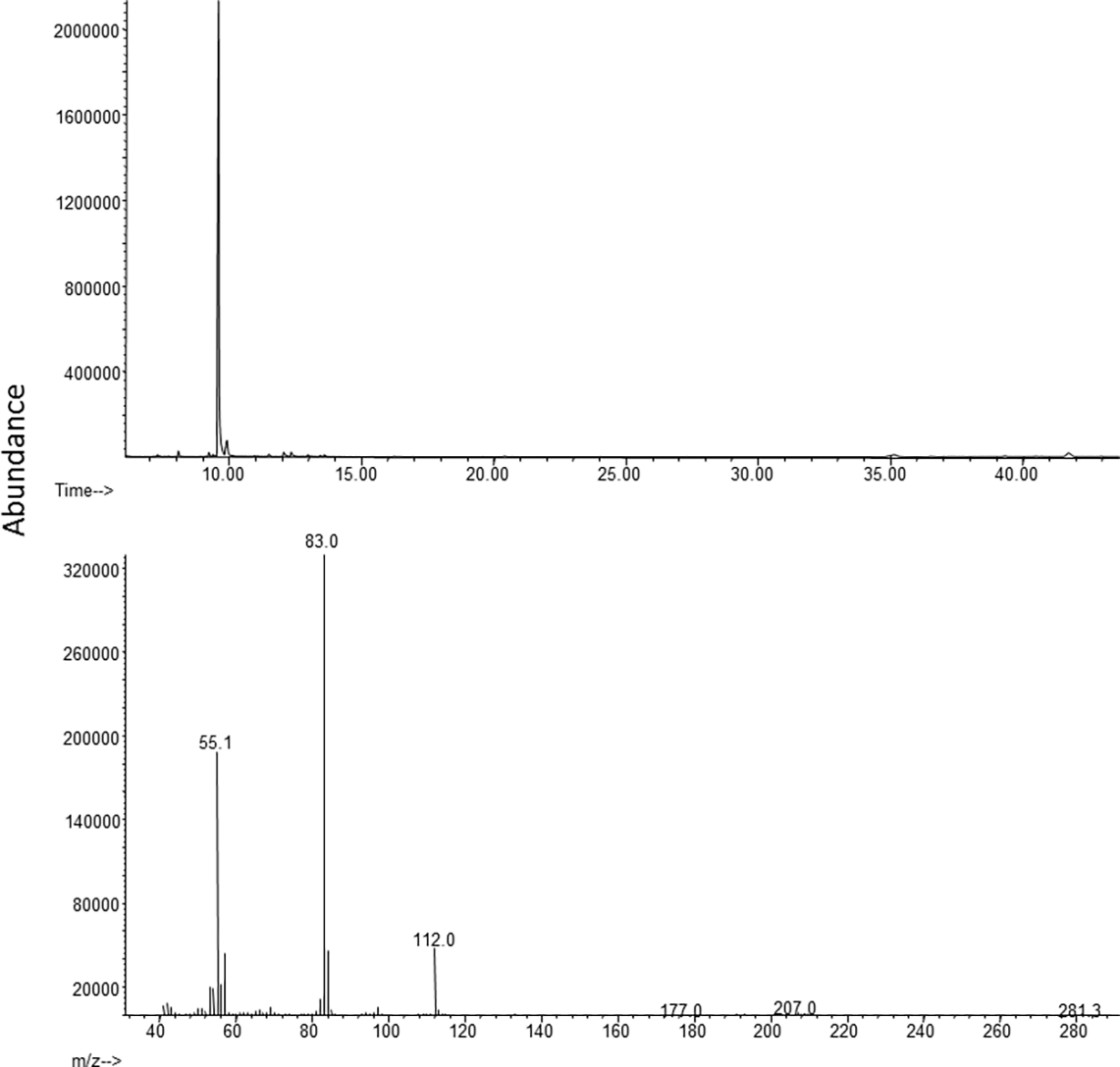
**A)** GC-MS total ion chromatogram and **B)** mass spectrum of synthetic as 4-oxo-(*E*)-2-hexenal.

The volatile 4-oxo*-(E)-*2-hexenal was identified by GC-MS (Fig 2) and detected as a major compound in all agitated samples. The retention time and the mass spectrum of the early eluting peak at 9.58 min were identical to those of the synthetic standard (Fig 3). Both C_6_ and C_8_ 4-oxo*-(E)-*2-alkenals are common within nymphal and adult Heteroptera secretions [30]. A minor component detected in four samples total was *(E)*-2-decenyl acetate.

The previously known and expected defensive compounds, *(E)*-2-octenal, *(E)*-2-decenal, and tridecane, were quantified Table 1). The amounts calculated to be released per adult of all three compounds did not follow a normal statistical distribution and there were no significant differences in absolute amounts between diapausing and diapause-disrupted adult *H*. *halys*: *(E)-*2-octenal (Mann-Whitney *U* = 3.0, n_1_ = 3, n_2_ = 3, *P* = 0.513), *(E)*-2-decenal (*U* = 23.0, n_1_ = 8, n_2_ = 6, *P* = 0.897), tridecane (*U* = 22.0, n_1_ = 8, n_2_ = 6, *P* = 0.796). This would suggest that adult *H*. *halys* produce and store the same amount of these compounds in their glands during diapause and active periods. Response ratios of these three compounds to the internal standard averaged at 0.32:1. All other compound amounts were therefore estimated assuming a 0.32:1 response ratio to the internal standard.

Gas chromatography-mass spectrometry is a well-established method for the detection and quantitation of volatiles, and appropriate for the work presented here. However, once VOC profiles have been confirmed, there are more novel techniques for detection of specified VOC profiles. Direct injection mass spectrometry (DIMS) methods, such as SIFT-MS and PTR-MS, are able to analyse ambient air samples in real time and cut out the need for an extraction step [31]. Both mentioned techniques use soft ionisation methods to minimise fragmentation of sample ions, reducing the number of ion overlaps on the resultant mass spectrum, which negates the need for chromatographic separation whilst still allowing complex VOC mixtures to be analysed in real-time [32]. Electronic noses have also been developed to detect pre-programmed VOC profiles in headspace. These devices benefit from being portable, they pull headspace from the area over carbon black-polymer composite sensors which are trained to a specific profile [33]. Studies have shown e-noses to exhibit up to 100% accuracy in detecting heteropterans, such as *Nezara viridula* and *Megacopta cribraria*, and damage caused by such species to host crops [33–35]. The disadvantage being that this device is still under recent development, and has been found to decrease in accuracy from 24 hours after training the sensors [33]. For the detection of *H*. *halys* emission profiles in contained spaces, both aforementioned could be valuable techniques to consider.

## Conclusions

The single compound, tridecane, consistently released by non-agitated diapausing adult *H*. *halys*, may be detectable, but it is not unique to *H*. *halys* or pentatomids. Such *n*-alkanes are commonly released biogenic volatile organic compounds. Instead, tridecane, *(E)*-2-decenal, 4-oxo*-(E)-*2-hexenal, and dodecane, should be considered collectively for a reliable emissions profile from both diapausing and diapause-disrupted adult *H*. *halys*.

For the context of chemical detection of *H*. *halys*, the aforementioned profile is relevant for particular scenario, and has the potential to be applied to large aggregations which have undergone mechanical agitation through transportation, such as the unloading of containers from ships. Were sufficiently sensitive detection methods to be developed, *(E)*-2-decenal could also be considered as an indicator compound for detecting general stink bug populations. Some novel VOC detectors known for specificity and sensitivity have been discussed. In addition, any detection method would also need to overcome the problem of background odors that may potentially interfere with the target volatilesIn terms of practical use, it will be necessary to know how such scenarios as freight and shipping affect diapausing *H*. *halys* behavior. Further studies are underway to assess the effects of movement and temperature fluctuation on odor emission under conditions resembling those on cargo ships.
